# Supplementary material for: The Clinical, Genomic, and Transcriptomic Landscape of BRAF Mutant Cancers
Source: Cancers (Basel). 2024 Jan 19;16(2):445. doi: 10.3390/cancers16020445 (PMC10814895; doi:10.3390/cancers16020445)
Supplement: Supplementary file 1 [file cancers-16-00445-s001.zip › Supplemental Figure and Table Legends.pdf]

Figure S1: Consort Diagram

Figure S2: The genomic landscape of BRAF mutant melanoma. A) Oncoprint highlighting the top 30 most frequent genes that are differentially altered between melanomas with Class 1/2/3 BRAF mutations. **B)** Histogram indicating the incidence of gene alterations within each BRAF class for the top 30 most frequently altered genes. **C)** The filtered list of genes that were significantly differentially altered according to BRAF Class 1/2 and 1/3 across all melanomas (n=31 and n= 153 respectively, Q<0.05) was subjected to pathway analysis using the using MSigDB Hallmark algorithm. All pathways that were over-represented in this list of genes are indicated in blue, red, and green (P<0.05 & Q<0.05, P<0.05 & Q<0.2, and P<0.1 & Q<0.2 respectively).

Figure S3: The genomic landscape of BRAF mutant Colorectal tumors. A) Oncoprint highlighting the top 30 most frequent genes that are differentially altered between tumors with Class 1/2/3 BRAF mutations. **B)** Histogram highlighting the incidence of gene alterations within each BRAF class. **C)** The filtered list of genes that were significantly differentially altered according to BRAF Class 1/2 and 1/3 status across CRCs (n=4 and n=44 respectively, Q<0.05) was subjected to pathway analysis using the using MSigDB Hallmark algorithm. All pathways that were over-represented in this list of genes (P<0.05 & Q <0.05) are indicated.

Figure S4: The genomic landscape of BRAF mutant NSCLC tumors. A) Oncoprint highlighting the top 30 most frequent genes that are differentially altered between tumors with Class 1/2/3 BRAF mutations. **B)** Histogram highlighting the incidence of gene alterations within each BRAF class. **C)** The filtered list of genes that were significantly differentially altered according to BRAF Class 1/2 and 1/3 status across NSCLC (n=6 and n=9 respectively, Q<0.05) was subjected to pathway analysis using the using MSigDB Hallmark algorithm. All pathways that were over-represented in this list of genes are indicated in red and blue (P<0.05 & Q<0.1, and P<0.1 & Q<0.1, respectively).

Figure S5: Gene ontology analysis on altered genes from all BRAF mutant cancer types. A. Top 18 altered genes were inputted for Gene Ontology Biological Process analysis (p<2.01 e-7, q< 0.001, hide gene-unconnected terms) B. Top 59 genes altered were inputted for Gene Ontology Biological Process analysis (p<2.13e-20, q<0.001, hide gene-unconnected terms).

Figure S6: **Violin plots displaying the distribution of age at sequencing according to BRAF Class** 1, Class 2, Class 3 and Fusion in all cancers, melanoma, CRC, non-small cell lung cancer (NSCLC) and other cancers (all cancers excluding melanoma, CRC and NSCLC). P-value was calculated using two-way ANOVA test for each table.

Table Legends

Table S1: Incidence and Classification of BRAF alterations BRAF Class was determined by protein changes shown in the table. The table describes the occurrence of each protein change from the 5120 BRAF mutant samples used for analyses.

Table S2: Co-Mutations across all cancer types in BRAF Class 1, 2 and 3 Genomic alterations for genes obtained from the GENIE v12 database for BRAF Classes 1, 2 and 3 within all cancer types. The table includes all listed genes, no modifications from the database.

Table S3: Co-Mutations across all cancer types in BRAF Class 1 and 2 Genomic alterations for genes obtained from the GENIE v12 database for BRAF Classes 1 and 2 within all cancer types. The table includes all listed genes, no modifications from the database.

Table S4: Filtered Co-Mutations across all cancer types in BRAF Class 1 and 2 Genomic alterations for genes obtained from the GENIE v12 database for BRAF Classes 1 and 2 within all cancer types. The table shows genes with minimum 15 samples mutated in BRAF Class 1/2, p<0.05, and q<0.05.

Table S5: Co-Mutations across all cancer types in BRAF Class 1 and 3 Genomic alterations for genes obtained from the GENIE v12 database for BRAF Classes 1 and 3 within all cancer types. The table includes all listed genes, no modifications from the database.

Table S6: Filtered Co-Mutations across all cancer types in BRAF Class 1 and 3 Genomic alterations for genes obtained from the GENIE v12 database for BRAF Classes 1 and 3 within all cancer types. The table shows genes with minimum 15 samples mutated in BRAF Class 1/3, p<0.05, and q<0.05.

Table S7: Co-Mutations across BRAF Class 1 and 2 melanomas Genomic alterations for genes obtained from the GENIE v12 database for BRAF Classes 1 and 2 within melanoma. The table includes all listed genes, no modifications from the database.

Table S8: Filtered Co-Mutations across BRAF Class 1 and 2 melanomas Genomic alterations for genes obtained from the GENIE v12 database for BRAF Classes 1 and 2 within melanoma. The table shows genes with minimum 15 samples mutated in BRAF Class 1/2, p<0.05, and q<0.05.

Table S9: Co-Mutations across BRAF Class 1 and 3 melanomas Genomic alterations for genes obtained from the GENIE v12 database for BRAF Classes 1 and 3 within melanoma. The table includes all listed genes, no modifications from the database.

Table S10: Filtered Co-Mutations across BRAF Class 1 and 3 melanomas Genomic alterations for genes obtained from the GENIE v12 database for BRAF Classes 1 and 3 within melanoma. The table shows genes with minimum 15 samples mutated in BRAF Class 1/3, p<0.05, and q<0.05.

Table S11: Co-Mutations across BRAF Class 1 and 2 colorectal cancers Genomic alterations for genes obtained from the GENIE v12 database for BRAF Classes 1 and 2 within melanoma. The table includes all listed genes, no modifications from the database.

Table S12: Filtered Co-Mutations across BRAF Class 1 and 2 colorectal cancers Genomic alterations for genes obtained from the GENIE v12 database for BRAF Classes 1 and 2 within melanoma. The table shows genes with minimum 15 samples mutated in BRAF Class 1/2, p<0.05, and q<0.05.

Table S13: Co-Mutations across BRAF Class 1 and 3 colorectal cancers Genomic alterations for genes obtained from the GENIE v12 database for BRAF Classes 1 and 3 within melanoma. The table includes all listed genes, no modifications from the database.

Table S14: Filtered Co-Mutations across BRAF Class 1 and 3 colorectal cancers Genomic alterations for genes obtained from the GENIE v12 database for BRAF Classes 1 and 3 within melanoma. The table shows genes with minimum 15 samples mutated in BRAF Class 1/3, p<0.05, and q<0.05.

Table S15: Co-Mutations across BRAF Class 1 and 2 NSCLC Genomic alterations for genes obtained from the GENIE v12 database for BRAF Classes

1 and 2 within melanoma. The table includes all listed genes, no modifications from the database.

Table S16: Filtered Co-Mutations across BRAF Class 1 and 2 NSCLC Genomic alterations for genes obtained from the GENIE v12 database for BRAF Classes 1 and 2 within melanoma. The table shows genes with minimum 15 samples mutated in BRAF Class 1/2, $p<0.05$, and $q<0.05$.

Table S17: Co-Mutations across BRAF Class 1 and 3 NSCLC Genomic alterations for genes obtained from the GENIE v12 database for BRAF Classes 1 and 3 within melanoma. The table includes all listed genes, no modifications from the database.

Table S18: Filtered Co-Mutations across BRAF Class 1 and 3 NSCLC Genomic alterations for genes obtained from the GENIE v12 database for BRAF Classes 1 and 3 within melanoma. The table shows genes with minimum 15 samples mutated in BRAF Class 1/3, $p<0.05$, and $q<0.05$.

Table S19: RNA expression Heatmap Genes CRC Gene list of RNA expression from BRAF mutant CRC heatmap. Genes are ordered from top to bottom as seen on heatmap. Cutoffs of baseMean>50, absolute Logfold>2, and padj<0.01 were used to obtain these genes. Log fold denotes the BRAF Class 1 vs BRAF Class 2&3 mutants.

Table S20: RNA expression Heatmap Genes NSCLC Gene list of RNA expression from BRAF mutant Non-Small Cell Lung cancer heatmap. Genes are ordered from top to bottom as seen on heatmap. Cutoffs of baseMean>50, absolute Logfold>2, and padj<0.01 were used to obtain these genes. Log fold denotes the BRAF Class 2&3 vs BRAF Class 1 mutants.

Table S21: RNA expression Heatmap Genes Melanoma Gene list of RNA expression from BRAF mutant Melanoma heatmap. Genes are ordered from top to bottom as seen on heatmap. Cutoffs of baseMean>50, absolute Logfold>2, and padj<0.01 were used to obtain these genes. Log fold denotes the BRAF Class 1 vs BRAF Class 2&3 mutants.
